# An Experimental Inquiry into the Proximate Cause of Fever

**Published:** 1824-05

**Authors:** R. Calvert


ART. IV.—*An Experimental Inquiry into the Proximate Cause of Fever.* By R. CALVERT, M.D.

UPWARDS of seven years ago I attempted to establish, by means of a small treatise, entitled "Reflections on Fever," what to me appeared to be a rational and consistent theory of febrile diseases. By availing myself of such facts and experiments as had then been promulgated, and by drawing my conclusions immediately from them, I was not without some hope of laying a foundation, upon which a permanent pathological and therapeutical structure would be raised.

But the mere pointing out of any particular doctrine, however sound in principle, at a time when the press is incessantly bringing forth new theories and systems, is a very inadequate means of insuring its acceptance by the medical republic; for "it is our firm conviction that, in medicine, as in religion, the great and fundamental truths must be not only brought forward, but *reiterated* from time to time, so as to be kept constantly before the eyes of the young, who are backward enough in learning them, and the old, who are forward enough in forgetting them."*

I shall here, then, recapitulate some of the leading points of my former doctrine, following them up by a series of statical experiments, which I have since instituted, for the purpose of filling up the spaces where I had employed the mere assertions of medical writers in place of authenticated facts.

The vascular system, I have said, is susceptible of considerable distension as well as relaxation; for, after death, several pounds of melted wax may be injected into it, in addition to its ordinary contents. During life and health there is a constant drain of fluids into and from this common mass; the ingress being chiefly through the thoracic duct, and the egress through the several excreting or secreting organs, as the skin, lungs, kidneys, liver, &c. Now, in order that a uniform quantity of fluids may be maintained in the system, the ingress and egress, in a given time, must be equal. This equilibrium I have denominated the *balance of circulation.*

Sanctorius, and his commentator Gorter, considered the

* *Medico-Chirurgical Review,* No. 6, p. 353.

standard of health to be marked by the equilibrium existing between the ingesta and the whole of the egesta; but, as the contents of the stomach and intestines are distinct from those of the vascular cavities, I exclude them from the system of circulation which I am considering.

It is quite evident that the balance of circulation may be lost in two different ways: viz. on the side of depletion, when the egress of fluids exceeds the ingress; or on the side of repletion, when the ingress exceeds the egress.

Depletion, again, may occur from diminished ingress; from augmented egress; or from both conjoined. Repletion, on the other hand, may arise from increased ingress; from diminished egress; or from both conjoined.

A febrile state of the body does not accompany depletion, as is proved by its not being immediately produced by abstinence, or by sudden evacuations from the vascular system, whether natural or artificial.

But we may observe, on the contrary, that a febrile state of the body is ever attended by repletion, the common indications of which are thirst, constipation of the bowels, dry skin, deficiency of urine, and the like. "It is to be particularly observed (says Dr. Cullen,) that, during the cold stage of fever, there seems to be a spasm induced every where on the extremities of the arteries, and more especially of those upon the surface of the body. This appears from the suppression of all the excretions."*

Now, every one must be aware that, if this statement of Dr. Cullen were literally true,—i. e. if a suppression of all the excretions took place while the usual intestinal absorption continued, the accumulated fluids would soon be sufficient to destroy the patient. They would amount, in fact, in a certain time, to as much as the patient would eat and drink.

In the absence of accurate information on this point, I have instituted the following experiments, in order to ascertain the precise state of the excretions during the progress of an intermittent, the purest example of febrile derangement.

The first was made on a gentleman of the commissariat department of the army, who had caught the disease (a tertian) while travelling in Greece. After having been free from it for some time, he suffered a relapse on the 9th May, 1816.

10th May, at a quarter before one o'clock P.M. and before dinner, he weighed *(nudâ personâ)* 137 lb. 4 oz.

11th May, at four P.M., when the paroxysm threatened to occur, as indicated by the usual feeling of lassitude, blueness of the nails, &c., and also before dinner, he weighed 137 lb. 14 oz., which was ten ounces more than on the preceding day of

* *First Lines*, par. 40.

intermission, at a quarter before one o'clock. The last paroxysm was arrested by a dose of opium.

Between the above-mentioned periods, a space of twenty-seven hours and a quarter, the patient's ingesta amounted to

| | | *oz. dec.* |
|---|---|---|
| | | 86.25 |
| Urine | 41.75 | |
| Fæces | 6.00 | |
| Weight gained | 10.00 | |
| | | 57.75 |

which leaves for pulmonary and cutaneous transpiration, 28.50

On comparing these results with others derived from experiments on a healthy subject, in the same island (Malta,) but at a time when the thermometer stood six degrees lower, the following discrepancies were observable:

| | | |
|---|---|---|
| Average ingesta in four consecutive days | | 96.75 |
| Urine | 52.75 | |
| Fæces | 8.62 | |
| | 61.37 | |
| To be deducted, Weight lost | 3.75 | |
| | | 57.62 |
| Transpiration | | 39.13 |

But, when the thermometer ranged from five to nine degrees higher, the average ingesta in six days was 80.08

| | | |
|---|---|---|
| Urine | 36.91 | |
| Fæces | 6.66 | |
| | 43.57 | |
| Weight lost | 7.11 | |
| | | 36.46 |
| Transpiration | | 43.62 |

On the 8th December, (one of the four days,) when the ingesta of this healthy subject amounted to 101 ounces, the wind being S.S.W. and the atmosphere foggy, the transpiration amounted to no more than twenty-six ounces. But it is to be remarked that the urine on that day weighed sixty ounces, so that the body gained no more than five ounces; and on no occasion did this healthy subject gain more than seven ounces in the space of twenty-four hours.

But neither the augmentation nor decrease of weight corresponded with the increase or decrease of food; for, when seven ounces had been gained in weight, no more than four ounces of food had been taken over and above the quantity of each of the two preceding days; and at another time, when the ingesta had increased from ninety-five to one hundred and eight ounces and a half, the body acquired no more than five ounces. But, on the following day, when the ingesta had been again reduced to one hundred and one ounces, an increase of other five ounces took place in the weight of the body.

The following are the results of an experiment performed on a patient labouring under a tertian. It commenced precisely with the beginning of a relapse.

| *Date.* | *Stage of Disease.* | *Hour.* | *Space.* | | *Weight.* | | *Ingesta.* | | *Urine and Fæces.* | | *Transpiration.* | | *Rate of Transpir. per Hour.* | | *Remarks.* |
|---|---|---|---|---|---|---|---|---|---|---|---|---|---|---|---|
| | | | *h.* | *m.* | *lb.* | *oz.* | *oz.* | *dec.* | *oz.* | *dec.* | *oz.* | *dec.* | *oz.* | *dec.* | |
| 1816. June 4th | Beginning of cold st. | $\frac{3}{4}$ p. 10 A.M. | | | 122 | 10 | | | | | | | | | Urine not evacuated. |
| | hot st. | $\frac{3}{4}$ p. 11 .... | 1 | 0 | 123 | 4 | 11 | 50 | | | 1 | 50 | 1 | 50 | |
| | sweating st. | $\frac{1}{2}$ p. 12.... | 0 | 45 | 122 | 4 | 4 | 50* | 22 | 50* | | | | | |
| | Continuation of do. | $\frac{1}{2}$ p. 3 .... | 3 | 0 | 121 | 11 | 20 | 75 | | | 29 | 75 | 9 | 91 | |
| | End of sweating st. | $\frac{1}{2}$ p. 7 .... | 4 | 0 | 121 | 14 | 24 | 50 | 11 | 50 | 10 | 00 | 2 | 50 | |
| — 5th | Intermission ...... | $\frac{1}{4}$ p. 8 A.M. | 12 | 45 | 120 | 3 | 28 | 50 | 28 | 00 | 27 | 50 | 2 | 11 | Urine evacuated. |
| | Ditto ............ | $\frac{1}{4}$ p. 12.... | 4 | 0 | 119 | 0 | 1 | 50 | 12 | 50 | 8 | 00 | 2 | 00 | |
| | Ditto ............ | 6 P.M.... | 5 | 45 | 122 | 15 | 78 | 50 | 4 | 00 | 11 | 50 | 2 | 00 | |
| — 6th | Beginning of cold st. | 8 A.M.... | 14 | 0 | 121 | 5 | 28 | 00 | 29 | 50 | 24 | 50 | 1 | 75 | Temp. of body, 97½°. |
| | hot st. | 9 A.M.... | 1 | 0 | 122 | 3 | 27 | 00 | 12 | 00 | 1 | 00 | 1 | 00 | Respirat. 40; Temp. 102°. |
| | Continuation of do. | 10 A.M.... | 1 | 0 | 122 | 0 | | | | | 3 | 00 | 3 | 00 | Temperature 103°. |
| | Begin. of sweating s. | $\frac{1}{2}$ p. 10.... | 0 | 30 | 121 | 12 | 6 | 00 | 9 | 25 | 0 | 75 | 1 | 50 | Respirations 26. |
| | End of do. ........ | $\frac{1}{2}$ p. 3 .... | 5 | 0 | 119 | 9 | 19 | 00 | 2 | 50 | 51 | 50 | 10 | 30 | Temperature 97°. |

* These two trials were entrusted to an assistant, who probably committed a mistake. Could the patient have absorbed two ounces from the atmosphere?

From the foregoing experiments, it appears that the human body, at the commencement of an intermittent paroxysm, is heavier, *cæteris paribus*, than during the intermission. In the last example, when the urine had not been evacuated, the body weighed, at the first febrile accession, 122 lb. 10 oz. But, if we deduct twenty-two ounces and a half, which were soon afterwards evacuated from the bladder, the absolute weight would have been 121 lb. 3½ oz.

On the morning of intermission, after the patient had taken a breakfast of twenty-eight ounces and a half, the urine having been evacuated, the body weighed only 120 lb. 3 oz. But, on the following morning, when the paroxysm recurred, *after eating only twenty seven ounces*, the urine having been again evacuated, the weight amounted again to 121 lb. 5 oz.

The absolute weight of this patient, then, was more than a pound greater at the commencement of the first paroxysm than it was, under similar circumstances, on the following morning of intermission; and from this period to that of his next attack, a space of twenty-three hours and three-quarters, the patient gained in absolute weight no less than eighteen ounces. The total ingesta during the latter space was one hundred and eight ounces, while the total egesta amounted to no more than ninety. The last table also shows the exact ratio in which the transpiration decreased in quantity, from the cessation of one paroxysm to the beginning of another.

It may be objected, that I have not yet proved that an accumulation takes place in the *vascular system*. I admit the defect: but, in absence of direct proof, may not thirst and constipation be considered as strong presumptive evidence of intestinal *dryness*, if I may be allowed the expression? and may not increased heat of the body, preceded and accompanied by hurried inspiration, be a sign of an augmented volume of blood, the *fons et erigo* of animal heat? At any rate, let the accumulation be where it may, I contend that it is more rational and philosophical to bleed and purge, and otherwise evacuate for a *redundancy* of humours, than to assign as reasons for so doing, like many modern pathologists, *increased action* or *excitement* in this and the other membrane, or *vascular congestion* in this or the other viscera.* Undoubtedly, inflammations and congestions do take place in many fevers; and local injuries have an unquestionable influence, through the nervous system or otherwise, in deranging the balance of circulation. But may not inflammation and congestion be sometimes the consequences of general distention, acting on comparatively weak, but healthy, parts; while, at other times, they may occur previous to febrile

* See the systems of Broussais, Clutterbuck, and others.

distention, from topical relaxation alone? The exanthematic fevers appear to be examples of the former occurrence, while pure traumatic fever seems to be indicative of the latter. But every modification of inflammation, I think, may be expected as each or both these causes (viz. general distention and local relaxation,) operate. What are usually denominated *active* and *passive* inflammations, may belong to the two extremes: that is to say, general distention may be essential in forming the active kind, while local relaxation may be more necessary in constituting the passive.

The above doctrine of accumulation is strikingly confirmed by its perfect agreement with the most respected, and indeed the only tangible, laws of therapeutics. For what are the means employed by nature to subdue febrile disorder, but those of evacuation? and what are those which imitative art prescribes, but such as evacuate the system, and restore within due limits the balance of circulation?

